# Complete Genome Sequence of Sinorhizobium meliloti Bacteriophage HMSP1-Susan

**DOI:** 10.1128/genomeA.01450-17

**Published:** 2018-05-03

**Authors:** Brennon Fleagle, Aldina Imamovic, Silvia Toledo, Madison Couves, Autumn Jensen, Mandy Vang, Ashley Steevens, Nevin D. Young, Michael J. Sadowsky, Betsy M. Martinez-Vaz

**Affiliations:** a Department of Biology and Biochemistry Program, Hamline University, Saint Paul, Minnesota, USA; b Department of Plant Pathology, University of Minnesota, Saint Paul, Minnesota, USA; c Department of Soil, Water and Climate, University of Minnesota, Saint Paul, Minnesota, USA

## Abstract

*Sinorhiozbium* bacteriophage HMSP1-Susan has a genome of 51,963 bp in size, with a GC content of 52.5%. It contains 97 putative coding sequences; 83% of these coding sequences (CDS) encode proteins classified as hypothetical or having unknown functions. HMSP1 has limited homology to previously reported viruses and likely represents a new phage that infects this nitrogen-fixing bacterium.

## GENOME ANNOUNCEMENT

The symbiotic relationships between bacteria of the genus Sinorhizobium (Ensifer) and legumes are important for sustainable agriculture because they facilitate the conversion of atmospheric dinitrogen to ammonia, which improves the overall productivity of crops (1). Despite the extensive body of knowledge on the molecular mechanisms that govern the establishment of nitrogen-fixing symbioses, little is known about the biotic factors that affect the survival of rhizobia in soil and their capacity to establish productive symbioses. Bacteriophages have been shown to select for specific types of Sinorhizobium strains and to affect the populations of nitrogen-fixing bacteria (2, 3). Currently, only five Sinorhizobium sp. bacteriophage genomes have been sequenced and reported in GenBank (4–8). Characterizing the genomes of Sinorhizobium phages is essential for understanding the contribution of viruses to the evolution of nitrogen-fixing bacteria and their role in the selection of genotypes associated with the establishment of optimal nitrogen-fixing symbioses (8).

Sinorhizobium meliloti bacteriophage HMSP1-Susan (referred to here as HMSP1) was isolated from agricultural soil in Salses, France (42°49'58''N, 02°55'08''E) and cultivated using Sinorhizobium meliloti strain HM007-12. Preliminary transmission electron microscopy examination revealed that HMSP1 has an icosahedral head of 60 nm width and a noncontractile tail of 100 nm length. This observation suggests that HMSP1 is a member of the Siphoviridae family of bacteriophages (9–11).

Total viral DNA was extracted using the Norgen Biotek phage DNA isolation kit (Thorold, ON, Canada). Genome sequencing was performed using the Illumina MiSeq v3 platform with single-read runs. The resultant 1,821,533 reads, with an average length of 151 bases and coverage equal to 5,227-fold, were successfully assembled into a single contig using a *de novo* assembly with the CLC Genomics Workbench program.

Phage HMSP1 has a linear double-stranded DNA genome of 51,963 bp and a GC content of 52.5%. Bioinformatics analysis of the genome ends and the protein sequence of the large terminase subunit showed that HMSP1 has a circularly permuted genome. Coding sequences within the HMSP1 genome were identified using GeneMark (12) and DNAMaster and annotated using BLASTp and PHASTER (13, 14). The phage genome encoded 97 proteins. These coding sequences (CDS) varied in length from 93 bp to 2,259 bp. From the total predicted putative genes, 62 are transcribed from the top (+) strand, and 35 genes are transcribed from the reverse (−) strand. HMSP1 did not contain any regions with significant similarity to noncoding RNA genes. Eighty-three percent of the proteins were classified as hypothetical or having unknown functions. The remaining 17% of the coding regions contained genes necessary for phage replication and assembly, including DNA helicases, DNA polymerases, and capsid, tail, and head proteins. Interestingly, HMSP1 encoded a putative chitinase and a putative DNA methylase with significant homology to proteins previously identified in Sinorhizobium and Geobacter phages, respectively. The results of BLASTn and ViroBlast analyses revealed that the HMSP1 genome did not have significant homology to previously reported bacteriophages; therefore, we propose that it represents a novel Sinorhizobium virus.

### Accession number(s).

The complete genome sequence of bacteriophage HMSP1-Susan has been deposited in GenBank under the accession number MG214783.
